# Harvester ant colonies differ in collective behavioural plasticity to regulate water loss

**DOI:** 10.1098/rsos.230726

**Published:** 2023-09-20

**Authors:** D. M. Gordon, E. Steiner, Biplabendu Das, N. S. Walker

**Affiliations:** ^1^ Department of Biology, Stanford University, Stanford, CA 94305, USA; ^2^ InfoGraphics Lab, University of Oregon, Eugene, OR, USA; ^3^ Hawai'i Institute of Marine Biology, University of Hawai‘i at Mānoa, Kāne'ohe, HI, USA

**Keywords:** collective behaviour, water scarcity, drought, long-term study, evolutionary rescue, behavioural variation among colonies

## Abstract

Collective behavioural plasticity allows ant colonies to adjust to changing conditions. The red harvester ant (*Pogonomyrmex barbatus*), a desert seed-eating species, regulates foraging activity in response to water stress. Foraging ants lose water to evaporation. Reducing foraging activity in dry conditions sacrifices food intake but conserves water. Within a year, some colonies tend to reduce foraging on dry days while others do not. We examined whether these differences among colonies in collective behavioural plasticity persist from year to year. Colonies live 20–30 years with a single queen who produces successive cohorts of workers which live only a year. The humidity level at which all colonies tend to reduce foraging varies from year to year. Longitudinal observations of 95 colonies over 5 years between 2016 and 2021 showed that differences among colonies, in how they regulate foraging activity in response to day-to-day changes in humidity, persist across years. Approximately 40% of colonies consistently reduced foraging activity, year after year, on days with low daily maximum relative humidity; approximately 20% of colonies never did, foraging as much or more on dry days as on humid days. This variation among colonies may allow evolutionary rescue from drought due to climate change.

## 1. Introduction

Behavioural plasticity, like plasticity in any phenotypic trait, is shaped by evolution, as reaction norms can be associated with fitness [1]. Behavioural responses can provide crucial adaptations to climate change and contribute to evolutionary rescue [2]. Intraspecific variation sets the potential for natural selection to shape adaptation in changing environments (e.g. [3–5]). As climate change accelerates, so do the evolutionary pressures to adapt to increasingly hostile environments [6–8]. To learn how behavioural plasticity contributes to adaptation to climate change, we need to examine behavioural variation among individuals, within populations, in their responses to changing conditions [9].

Most studies of behavioural plasticity focus on individuals, but groups also show plasticity in collective behaviour. For example, social groups of baboons differ in space use and in interactions with neighbouring groups [10]. Social insect colonies provide an opportunity to learn about the plasticity of collective behaviour and how it varies [11]. Colony activity is regulated collectively, allowing the colony to adjust to changing conditions [12]. From an evolutionary perspective, the behavioural plasticity of a social insect colony can be considered to be the plasticity of an individual, because a colony functions as a reproductive individual. A colony includes one or more mated female reproductives who produce many sterile female workers, female reproductive daughters, and male reproductive sons. Both workers and reproductives contribute to the survival of the colony and the reproduction of offspring colonies. New offspring colonies are founded after mating between male and female reproductives of different colonies. Thus each colony is the offspring of parent colonies and may in turn contribute offspring colonies to the next generation.

Just as individuals differ in behavioural plasticity, so do social insect colonies, for example in foraging and exploration, in ants [13–16], honeybees [17–20], bumblebees [21], hornets [22] and other wasps [23]. When such variation is associated with colony reproductive success [19,23], this can provide the raw material for natural selection [24–27].

The red harvester ant *Pogonomyrmex barbatu*s lives in desert grassland and eats the seeds of annual plants and grasses. Colonies regulate foraging activity in response to water stress [25,28,29]. Desiccation is an important environmental pressure for desert organisms [30], and drought induced by climate change increases this pressure [31]. Harvester ant foragers lose water to evaporation while out in the sun searching for seeds, and obtain water from metabolizing the fats in the seeds that they eat. Thus colonies must spend water to obtain water and food.

Harvester ant colonies forage for seeds scattered by wind and flooding [32]; unlike other ant species that recruit to ephemeral or patchy resources, they do not use pheromone trails to recruit others to a particular site. Foragers travel in a stream from the nest, and then each forager leaves the trail to search for seeds. On repeated trips in the course of a day, a forager returns to search at the same site [33]. The directions that foragers take are set each morning by a small group of workers, the patrollers, apparently in response to encounters with neighbouring conspecific colonies [34], which compete for foraging area [35,36].

Colonies differ in the timing and extent of foraging activity each day [13]. In the summer, foraging begins soon after sunrise and ends at approximately noon [37] when ground temperature rises above approximately 50°C and humidity declines. Foraging activity depends in part on food availability. When a forager returns to the nest, it puts down the food it brought back in the entrance chamber just inside the nest entrance. Whether the forager leaves on its next trip depends on the rate at which it encounters, through antennal contact, returning foragers bringing in food [38–41]. The rate of forager return is related to food availability, because each forager searches until it finds a seed and then immediately returns to the nest, so the more food is available, the more quickly foragers return. Colonies vary in how outgoing foragers respond to the rate of forager return [14,41,42].

On the day-to-day timescale, foraging activity is adjusted in response to humidity, as in other seed-eating species in arid climates (e.g. [43,44]). Temperature and humidity conditions in the entrance chamber remain constant [29], so a forager can assess conditions outside only when it leaves the nest; its experience on one foraging trip can influence its decision whether to leave again on its next trip. During the summer monsoon season in the southwest USA, some days are overcast or rainy, while others are hot and dry. In general, colonies tend to forage more on humid days. For example, a study of 17 colonies over 3 years (2009–2011) showed that colonies tended to make fewer foraging trips on dry days with a lower vapour pressure deficit as measured by dew point [28].

Colonies differ in their responses to day-to-day changes in humidity [25]. Within a given year, some colonies consistently reduce foraging activity on dry days, thus sacrificing food intake and conserving water, while other colonies do not [28,45]. These differences among colonies in how they regulate foraging activity in dry conditions are associated with physiological differences. First, workers of colonies that reduce foraging on dry days showed lower tolerance for water stress than those of colonies that do not reduce foraging on dry days [46], perhaps due to colony differences in cuticular hydrocarbons, which provide waterproofing [47,48]. Second, colony differences in the propensity to reduce foraging in dry conditions are also associated with the expression of genes associated with dopamine metabolism [49], and in increased foraging activity when fed dopamine in field experiments [50].

These physiological differences among colonies, associated with how they adjust foraging in dry conditions, are apparently common to all individuals within a colony. Since temperature and humidity remain constant inside the nest while changing outside throughout the day [29], a forager's assessment of current humidity must depend on the water loss it experienced outside on previous trips. A study with uniquely marked individuals showed that the foragers in a particular colony share a threshold at which it has become too dry to forage at the end of the morning activity period [51].

How a colony regulates foraging activity in response to dry conditions is a crucial behavioural reaction norm [52] that can influence its survival and reproductive success. A previous study showed that colonies that reduce foraging in dry conditions, sacrificing food intake to conserve water, show higher reproductive success, in offspring colonies, than those that do not [25]. If this collective behavioural plasticity is heritable, it can be shaped by natural selection. This may become increasingly important, as drought conditions in the southwest USA are predicted to grow more severe [53,54].

The first step in learning how collective behavioural plasticity is evolving in a natural population is to determine what variation is present that selection can act upon (e.g. [55]). Here we extend previous work on colony differences in response to day-to-day changes in humidity, measured within a given year, to ask whether these differences among colonies are consistent from year to year. We draw on a longitudinal study of a population of colonies to ask: (i) whether there is an absolute threshold level of humidity, shared by all colonies, that tends to trigger a reduction in foraging activity every year, and (ii) whether there are persistent differences among colonies, year to year, in how they reduce foraging on dry days.

## 2. Methods

### 2.1. The study population

We measured the foraging behaviour of colonies of the red harvester ant, *Pogonomyrmex barbatus,* at the site near Rodeo, New Mexico, USA of a long-term study that has monitored this population, approximately 300 colonies per year, since 1988 [36]. The ages and locations of all colonies are known through an annual census in which approximately 1500 colonies have been monitored throughout their lifetimes.

### 2.2. Measures of foraging activity

Foraging behaviour was measured each year in each colony for 7–11 days that spanned a range of humidities. Observations were conducted in August in each of the 5 years from 2016 to 2021, excluding 2017 (table 1). Most colonies were reproductively mature, 5 years or older [36]. Each year after 2016 we measured foraging activity in all of the colonies measured previously that were still alive. The total number of colonies observed each year (with the number of these that were of age less than 5 in parentheses) was: 2016: 29(2), 2018: 58(18), 2019: 108(24), 2020: 82(20), 2021: 85(29). There were 93 colonies observed in at least 2 years, on 3514 colony-days. Of these colonies, 42 were observed in 2 years, 33 in 3 years, 17 in 4 years, and 1 in all 5 years. Each year we also added some new colonies, chosen based on location rather than any evaluation of foraging behaviour, choosing the colonies closest to others being counted so as to facilitate rapid counts. The numbers of colonies observed only in 1 year, because of colony death or a location incompatible with rapid counts, were: 2016: 10(1), 2018: 35(2), 2019: 27(5), 2020: 13(5), 2021: 21(19). Of these 106 colonies, 12 were not active on enough days to be included in data analysis (see below), so the analysis included 94 unique colonies observed foraging actively on a total of 880 colony-days. Table 1 shows the number of colonies observed to forage actively and the daily maximum humidity on each day for each year; daily values for other measures are shown in electronic supplementary material, table S1.

**Table 1.** Summary of data. The columns show the year and dates in August when foraging counts were made; the number of colonies observed foraging actively that day; the mean foraging count, in numbers of ants travelling to and from the nest in 30 s, for all colonies observed that day, and the daily maximum relative humidity (%) that day.

year	date	number of colonies foraging	mean foraging count per colony (foragers/30 s)	daily maximum RH (%)
2016	18	25	10	47
	19	28	15	67
	20	26	18	88
	22	29	31	85
	23	29	27	79
	24	28	30	67
	25	28	34	71
	26	27	35	83
	27	29	36	88
	29	29	24	69
	30	29	25	72
2018	16	75	62	88
	17	98	45	78
	18	98	31	73
	20	96	45	81
	21	95	46	78
	22	96	46	89
	23	98	53	89
	24	97	35	94
	25	97	68	95
	28	98	20	76
2019	17	96	34	67
	18	98	40	78
	19	104	43	68
	21	105	30	51
	22	107	49	75
	23	107	54	92
	24	107	49	81
2020	20	67	50	66
	21	70	54	75
	22	78	60	86
	24	79	66	46
	25	81	68	44
	26	82	60	35
	27	81	71	44
	28	79	65	50
2021	19	75	33	96
	20	78	26	90
	21	85	30	86
	23	85	54	86
	24	85	50	93
	25	85	50	81
	26	85	49	74
	27	85	41	64
	28	85	46	74
	30	85	43	92
	31	85	48	94

Foraging activity begins early in the morning with a wave of foragers leaving the nest [29]. After the initial surge of ants leaving the nest, foraging activity reaches a steady state in which the rate at which foragers leave the nest is approximately equivalent to the rate at which they return [29]. To evaluate day-to-day changes in foraging activity, we measured foraging activity when colonies reached a steady state, with approximately equal numbers leaving and returning, usually between approximately 8.00 and 9.00, but ranging from 7.30 to 11.00 depending on weather conditions. We attempted to count all colonies as rapidly as possible so as to obtain counts of all colonies in similar conditions. Each observer made counts in a set of approximately 20 nearby colonies per day, and each day reversed the sequence of counts so that certain colonies would not be counted only at the beginning or end of the observation period.

Foraging activity was measured as in previous work [51]. For each colony, on each day, we counted the number of incoming and outgoing foragers crossing an imaginary line perpendicular to the trail, for each foraging trail or direction, in 30 s. Foraging rate was the sum or total number of foragers moving toward or away from the nest in 30 s. The number of foraging directions a colony takes on a given day can range from one to six, and sometimes, in colonies in areas of sparse vegetation, the ants fan out radially from the nest mound [35,42]. If the ants were dispersed rather than in a trail, we defined up to six sectors of an imaginary circle around the nest mound and counted ants crossing an arc across the outer edge of that sector. Counts were made at least 1 m from the edge of the nest mound to ensure that ants of other task groups working on the mound, such as nest maintenance workers [56], were not counted as foragers.

A colony of *P. barbatus* does not forage actively every day [25,42]. We excluded from the data analysis the 504 observations from days when the colony did not forage at all, leaving a total of 3010 colony-days of observations of actively foraging colonies. On the days when a colony did not forage, either there were no ants active outside the nest at all, or there were some ants active, for example nest maintenance workers bringing out refuse, but no ants were foraging. The first situation, when no ants come out at all, is apparently not related to current humidity, as the ants are too deep inside the nest, where humidity conditions are constant [29], to assess humidity conditions outside the nest. Observations inside the nest with a videoscope show that on days when there is no activity outside the nest, ants do not come up from the deeper nest to the entrance chamber just inside the nest entrance. Observations suggest that a colony is not active at all when it has collected a large amount of food in the previous few days. In the second case, small numbers of ants leave the nest early in the foraging period, to do patrolling and nest maintenance work such as carrying out refuse, but then the colony does not go on to forage that day.

### 2.3. Weather data

We asked how foraging activity was adjusted in response to the day-to-day changes in humidity that influence water loss by foragers. Vapour pressure deficit, which determines evaporative water loss, depends on both temperature and humidity. From day to day in the summer, humidity varies more than temperature. Depending on whether the day is overcast or sunny, humidity differs even though temperatures remain high.

We used temperature and relative humidity data provided in approximately 5 min increments from the New Mexico Rodeo Airport Station (31.95° N, 109.05° W, https://www.wunderground.com/dashboard/pws/KNMRODEO1) for all years, except for 1 day for which data were not available. Data for this day, 26 August 2020, were obtained from Stone Hill Station, AZ (31.90° N, 109.11° W, https://www.wunderground.com/dashboard/pws/KAZSANSI27).

### 2.4. Differences among days in conditions

We chose daily maximum relative humidity as the measure of conditions to use in further analysis, based on the following procedure. Evaporative water loss depends on vapour pressure deficit, a function of both temperature and relative humidity [57]. We do not know what features of humidity conditions the ants respond to, and what is the lag between the change in condition and a change in foraging activity. Previous work suggested that colonies adjust foraging activity in relation to mean daily dew point, a measure that combines temperature and humidity [28]. To examine the relation between foraging activity and conditions, we explored 17 different measures of humidity and temperature on the daily and hourly timescale (electronic supplementary material, table S2).

First, we found in each year the driest and most humid days according to each measure. To do this, we ranked the days, found the quartiles and designated the lowest and highest quartiles as the dry and most humid days for that measure in that year. When ranks were tied, we arbitrarily chose a day.

We then determined whether foraging activity differed significantly on the most humid and dry days using a given measure. We performed a permutation test to compare the differences in foraging activity between the most dry and most humid days with the differences between a random selection of dry and humid days. We generated 1000 permutations of randomized hypothetical ‘humid’ and ‘dry’ days, by assigning a random number to each day of observation within a year, and rank ordered these. We found the top and bottom quartiles of the ranked list, and we balanced the number of days within each year to have the same number of humid and dry days as the measures for that year. We found the per cent reduction in numbers foraging between the top relative to the bottom quartiles. We used a Kolmogorov–Smirnov test to compare the per cent reduction in foraging using the most dry and most humid days with the difference for randomly chosen dry and humid days. The distribution differed significantly from the random assignment of humid and dry days for all measures except the range of humidity during the morning foraging period ((Kolmogorov–Smirnov, *D* = 0.05, *p* = 0.14), electronic supplementary material, table S2). To check the effect of choosing a day at random when ranks were tied, we performed the same test choosing the other tied day, and the results were the same.

**Figure 1.**
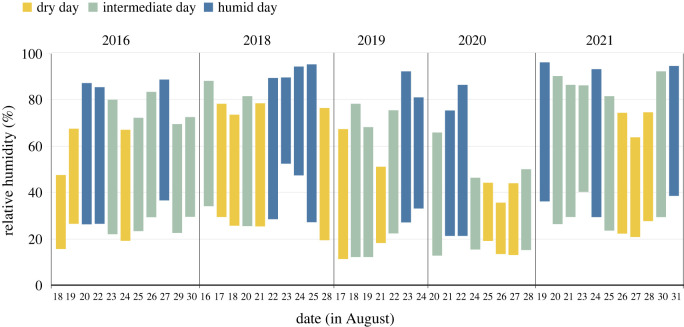
Days designated as most dry and most humid by year, using the daily maximum relative humidity. Each bar shows the range in relative humidity each day that foraging activity was observed, and the colours designate the 2 or 3 days chosen as the most dry or most humid each year: yellow for dry days, blue for humid days and light green for other days.

For further analysis, we used daily maximum relative humidity (figure 1). This measure gave the lowest *p*-value in the permutation test (electronic supplementary material, table S2), thus distinguishing foraging activity on dry and humid days most, compared with a random assignment of days as dry or humid.

#### 2.4.1. Do colonies adjust foraging activity to a relative or absolute threshold in humidity?

For each year, we used the measure of foraging activity (total foraging rate in 30 s) for all colonies on all 7–11 days observed that year, including both the colonies monitored longitudinally and those observed only in that year, to ask how the relationship between foraging activity and daily maximum relative humidity changes in response to conditions that year. Humidity conditions differed from year to year (table 1, figure 1). For each colony in a given year, we normalized foraging activity for colony size as a proportion of the maximum foraging activity measured in that colony in that year. We used a generalized linear mixed effects regression model (GLMM) using the lme4 package for R [58] with normalized foraging rate as the response variable. For the full model, we used daily maximum relative humidity, year and an interaction between humidity and year as fixed predictor variables, and colony ID as an intercept-only, random effect. To determine whether the relation of humidity and foraging differed among years, we compared this full model with one without the interaction term. The relation of foraging and humidity differed among years, so we further tested whether foraging activity depended on daily maximum relative humidity in that year. For each year, we compared the model with humidity as the only fixed effect and the intercept for colony ID as a random effect with one with no fixed effect and the intercept for colony ID as a random effect. We used *χ*^2^ tests to assess significance.

#### 2.4.2. Is there consistent variation among colonies in tendency to reduce foraging when dry?

We developed an index to characterize differences among colonies in how they adjust foraging activity in dry conditions, so as to determine whether these differences among colonies persisted over the 2–5 years that colonies were observed. We used this index to distinguish two groups of colonies, those that consistently reduce foraging when dry, and those that consistently do not. We then tested whether the adjustment of foraging activity to humidity was significantly different in the two groups of colonies.

First, to characterize a colony's response to dry conditions, for each colony and each year observed, we compared its foraging activity on the driest and most humid days of that year (figure 1). In each year we compared a colony's foraging activity in the same number of dry and humid days, either 2 or 3. If a colony was not active at all on some of the dry or humid days, but was active on at least one each of the dry and humid days, we averaged the foraging counts for the days when it did forage actively. If a colony was not active at all on any of the dry or any of the humid days, we did not calculate the index for that colony for that year. As a result, the numbers of colonies included in the data analysis are lower than the total numbers observed.

Because we were comparing counts for the same colony on the driest and most humid days within 7–11 days (table 1), not comparing across colonies, we used the raw counts of foraging rate each day and did not normalize for colony size. (An interval of 7–11 days is too brief for significant changes in the size of a given colony due to worker production.)

For each year and each colony, we calculated two measures of the difference in a colony's foraging rates between the most humid and most dry days, using daily maximum RH. First, for each colony, we found the proportion by which foraging activity was reduced on dry days, relative to humid days, as the ratio of foraging activity on dry days to foraging activity on humid days. Second, for each colony, we found the difference between this proportion and the median proportion for all colonies in that year.

We categorized a colony in a given year as one that reduced foraging in dry conditions when the proportion reduction in foraging activity on dry days, relative to humid days, was at least 20%. We chose 20% based on the distribution of reduction of foraging (figure 5); most colonies that reduced foraging in a given year did so less than 20%, except for in 2018 (figure 5*a*). For the second measure, we categorized a colony in a given year as one that reduced foraging when dry when the proportion reduction in foraging activity on dry days, relative to humid days, was below the median for all colonies that year. The two measures led to similar results in sorting colonies into those that consistently reduced foraging on dry days, or did not, from year to year. We used the proportion reduction with a cutoff of 20% in subsequent analyses.

We considered a colony to reduce foraging in dry conditions when it did so in most of the years observed: 2 of 2, at least 2 of 3, and at least 3 of 4 or 5 years, equivalent to a threshold of 60%.

We then used two methods to examine differences among colonies in how they adjust foraging activity in dry condition. First, we compared the relation of foraging activity and humidity in two sets of colonies: (i) the 35 colonies observed in two or more years that were identified to reduce foraging in dry conditions, and (ii) the 94 colonies that were observed only in one year and thus had not been categorized as either reducing foraging or not when dry. Whether a colony was observed in only 1 year was unrelated to its foraging behaviour, so the observations from the second set of 94 colonies provided a group for comparison with colonies that consistently reduce foraging on dry days. We found for each set of colonies the distribution of reduction in foraging, either the proportion reduction on dry days relative to humid days or the difference from the median reduction in foraging. We compared the distributions of the two sets using a Kolmogorov–Smirnov test.

Second, we compared the adjustment of foraging activity to humidity in the 35 colonies that consistently reduced foraging on dry days and the 72 colonies that consistently did not, using a GLMM following the methods of Schielzeth and Nakagawa [59] using R [60]. As in Araya-Ajoy *et al*. [61], we treated humidity conditions as a categorical variable, assigning to each day a value for dry (−1), intermediate (0), and humid (1) as described above. Because this measure was calculated separately for the range of humidity within each year, and thus normalized for differences among years, we included data from all days and all years. The response variable was foraging rate, normalized for colony size as in §2.4.1 above, as a proportion of the maximum foraging activity measured in that colony in that year. Fixed predictor variables were the additive effects of humidity level (dry, intermediate and humid), whether the colony reduced foraging on dry days or not, and the slope for humidity level and intercept for colony ID were random effects. The full model was [foraging ∼ humidity level × reducerYN + (1 + humidity level | colonyID)]. To determine whether the relation of foraging activity and humidity level differed among colonies within the two groups, those that reduced foraging on dry days and those that did not, we first compared the full model to one without the random slope for humidity level, using a *χ*^2^ test. Next, to determine whether the two groups of colonies, those that reduce foraging on dry days and those that do not, differ in the slope of foraging versus humidity level, we compared the full model with one without the interaction term, using a *χ*^2^ test. We then used Wilcoxon rank sum tests to compare foraging activity at each level of humidity (dry, intermediate and humid) in the two sets of colonies, those that reduced foraging in dry conditions and those that did not.

## 3. Results

### 3.1. Do colonies adjust foraging activity to a relative or absolute threshold in humidity?

**Figure 2.**
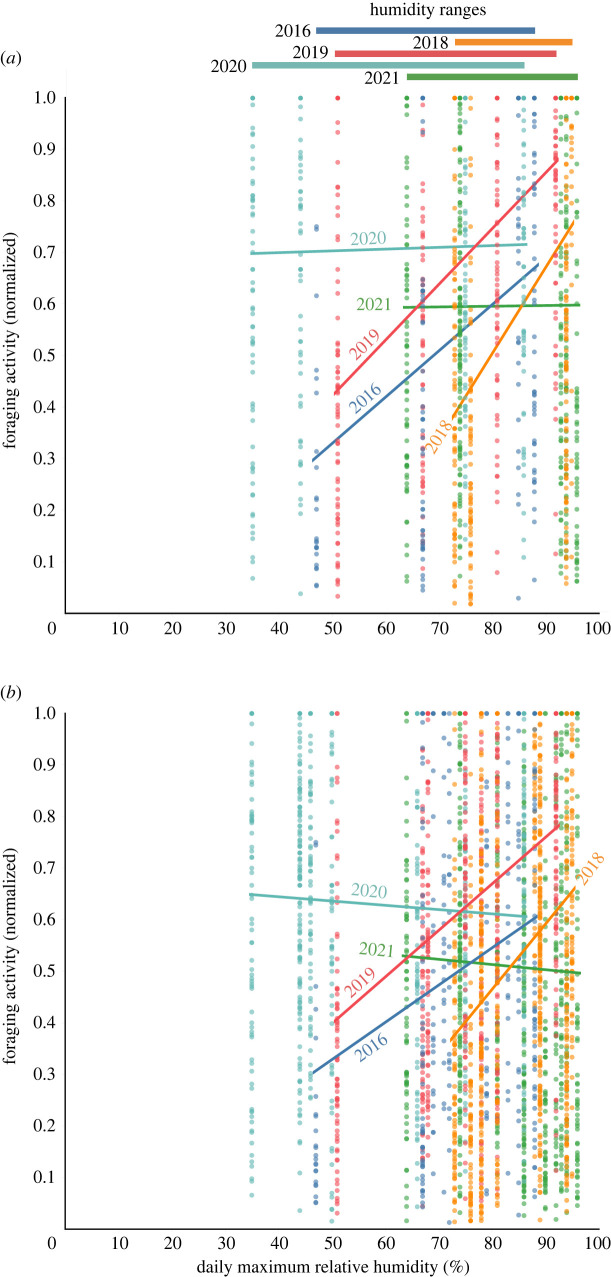
Relation of foraging activity and humidity by year. Each point represents foraging activity for one colony on one day, normalized for colony size as the proportion of the largest number of ants seen foraging in that colony in that year. (*a*) Data are shown for all colonies and all days of observation each year. (*b*) Data are shown only for the most dry and humid days each year. The range of relative humidity observed each year is shown above the graph. The lines show the least-squares fit of foraging activity on daily maximum relative humidity for each year.

The relation of foraging activity to humidity varied from year to year (interaction of year and humidity: *χ*^2^ = 199.8, *p* < 0.001). The years differed greatly in the range of humidity observed, and in the extent to which colonies adjusted foraging activity to humidity (figures 2 and 5*a*). In some years (2016, 2018, 2019), the general trend was for colonies to reduce foraging on dry days, but in other years (2020, 2021), there was no trend when considering the behaviour of all colonies observed that year. The magnitude of the decrease in foraging on dry days, relative to humid ones, varied from year to year (figure 5*a*). Colonies were more likely to decrease foraging on dry days, relative to humid days, in 2016, 2018 and 2019 than in 2020 or 2021. Statistical analysis confirmed that foraging activity was significantly reduced on dry days in some years but not in others. Foraging activity depended significantly on daily maximum relative humidity in 2016 (*χ*^2^ = 29.4, *p* < 0.001), 2018 (*χ*^2^ = 143.9, *p* < 0.001) and 2019 (*χ*^2^ = 133.7, *p* < 0.001), but not in 2020 (*χ*^2^ = 0.5, *p* = 0.5) or 2021 (*χ*^2^ = 0.3, *p* = 0.6).

**Figure 3.**
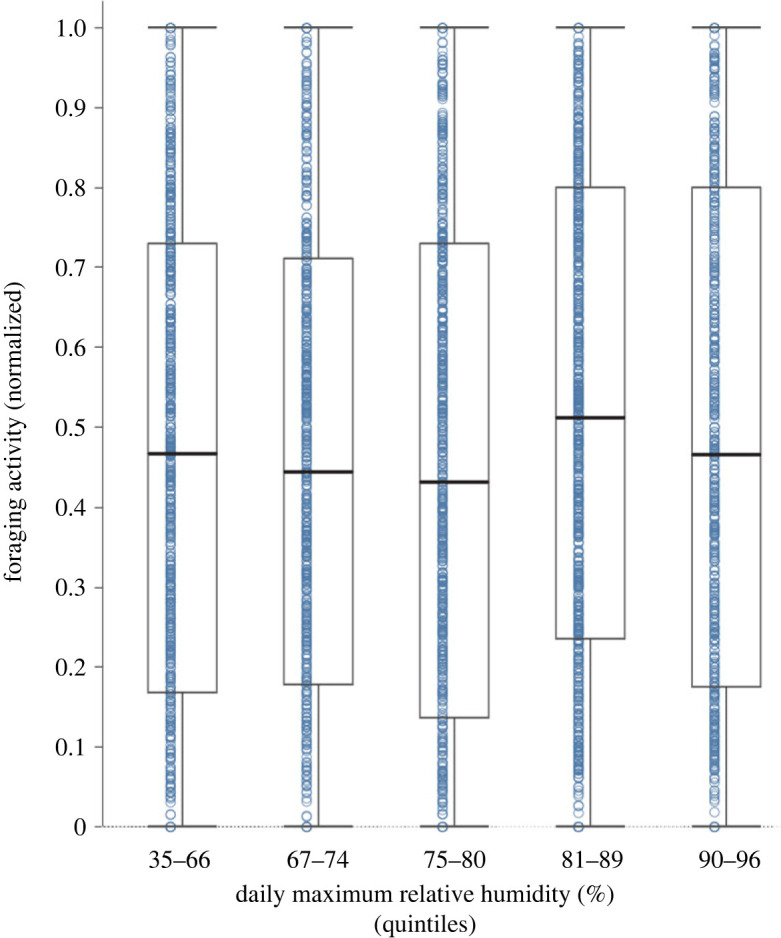
Foraging activity by humidity. Data are shown for all colonies observed in all years. Each point represents foraging activity for one colony on one day, normalized for colony size as the proportion of the largest number of ants seen foraging in that colony in that year. On the x-axis, all values of daily maximum relative humidity are grouped by quintile. The values are the bottom and top for each quintile; for example, the lowest 20% of daily maximum relative humidity observed in any year ranged from 35% to 66%.

There is no evidence of an absolute threshold below which colonies tend to decrease foraging activity in all years. Instead, colonies adjust foraging in relation to recent humidity levels in the current year rather than to any absolute value of humidity common to all years. When data from all colonies and all 5 years were combined, foraging activity was approximately the same regardless of humidity (figure 3). Figure 2 also shows that from year to year, colonies adjust foraging activity to humidity at different absolute values of humidity. For example, in 2018, when daily maximum relative humidity was high, ranging from approximately 70% to 95%, many colonies reduced foraging at the low relative humidity for that year of 70%, while in 2016, when relative humidity ranged from 40% to 85%, foraging was high at 70% and reduced foraging only at the low humidity for that year of 30–40%.

### 3.2. Is there consistent variation among colonies in tendency to reduce foraging when dry?

Some colonies consistently reduce foraging on dry days, year after year, while some consistently do not, and others do so only rarely. Out of 93 colonies observed in at least 2 years, 35 (38%) consistently reduced foraging on dry days, with foraging activity at least 20% lower on dry days, with low daily maximum relative humidity, relative to humid days, with high daily maximum relative humidity, in most years observed (greater than 60% of years observed, i.e. 2 out of 2, at least 2 out of 3, and at least 3 out of 4 or 5 years). Using the difference from the median proportion reduction on dry days relative to humid days, 27 (29%) of the 93 colonies reduced foraging more than the median proportion reduction for that year. There were 19 colonies that consistently reduced foraging on dry days in most years for both measures, 18 more that did so for the 20% measure only, and another eight that did so for the median measure only.

Of the remaining colonies that were observed in more than one year, 22 (24%) never reduced foraging by more than 20% on dry days or did so in less than a third of the years they were observed, and 35 (38%) colonies did so in less than 50% of the years they were observed.

**Figure 4.**
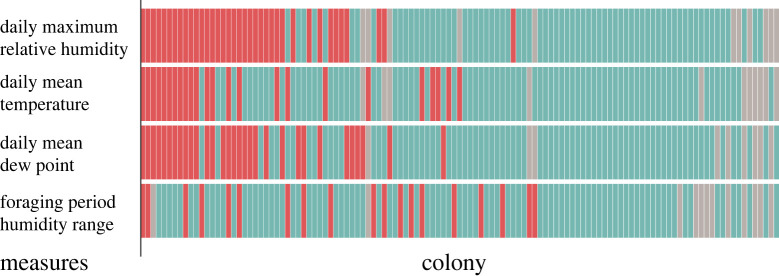
Differences among measures in year-to-year persistence of collective behavioural plasticity. Each column shows data for one colony. Each row shows the results for one measure of humidity, temperature or both. Red indicates a measure for which a colony reduced foraging on dry days, relative to humid days, by at least 20%, in more than half of all years observed; blue indicates a measure for which a colony did not reduce foraging on dry days in most years, and beige indicates a measure for which there were insufficient data for at least 2 years, because the colony did not forage actively on enough dry and humid days.

More colonies were consistent from year to year in reducing foraging activity on dry days, measured by daily maximum relative humidity, than in other measures of temperature or humidity. Figure 4 shows, for each colony, whether it consistently reduced foraging on dry days according to several measures of humidity, temperature or both. Consistency in foraging response to dew point was most similar to response to daily maximum humidity, while response to temperature alone was much less consistent. The fewest colonies were consistent from year to year in reducing foraging activity on dry days as measured by the range of humidity during the morning foraging period, which, as described above, did not yield a significantly different pattern of foraging activity from randomly selected days.

**Figure 5.**
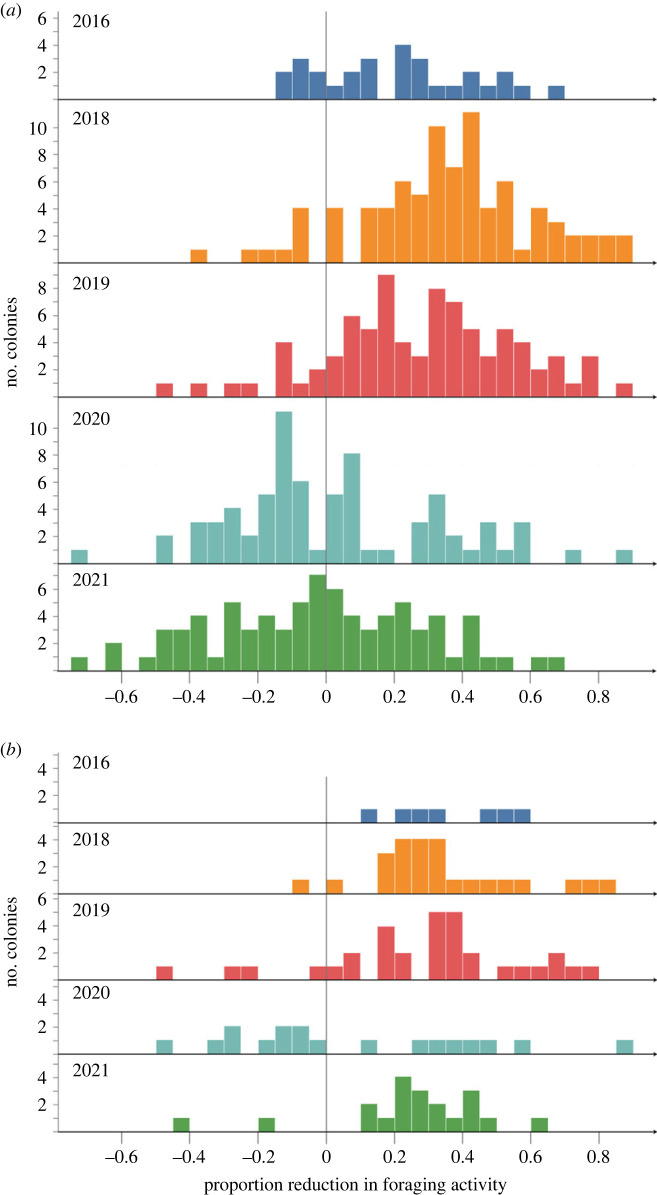
Frequency distributions for each year of the proportion reduction in total foraging activity on the driest days, relative to the most humid days, measured using daily maximum RH. The bars on the right, with positive values for the proportion, show the number of colonies that reduced in foraging on dry relative to humid days; the bars on the left, with negative values, show an increase in foraging on dry days relative to wet days. (*a*) Data are shown for all colonies. (*b*) Data are shown only for colonies that consistently reduced foraging on dry days in more than 60% of years observed.

The distribution of reduction in foraging for all colonies observed each year, comparing the days with lowest and highest daily maximum relative humidity, is shown in figure 5*a*. Some colonies reduce foraging on dry days, some do not, and some even increase foraging on dry days relative to humid ones (figure 5*a*). The same distribution is shown in figure 5*b* only for the subset of colonies that reduced foraging by more than 20% in most years observed. The distribution in figure 5*b* is clearly skewed more to the right, representing a reduction in foraging by this subset of colonies compared with the distribution for all colonies shown in figure 5*a*.

The colonies that consistently reduced foraging on dry days, using the daily maximum relative humidity, in most (greater than 60%) of the years observed, did so significantly more than the set of 94 colonies observed only in one year. The distribution of the proportion reduction in foraging for the 37 colonies that reduced foraging on dry days by more than 20% in most years observed was significantly different from the distribution for the 94 colonies observed in a single year (Kolmogorov–Smirnov test, *D* = 0.19, *p* = 0.03). The distribution of the difference from the median reduction in foraging for the 27 colonies that reduced foraging on dry days on most years observed was also significantly different from the distribution for those observed only in one year (Kolmogorov–Smirnov, *D* = 0.22, *p* = 0.02).

**Figure 6.**
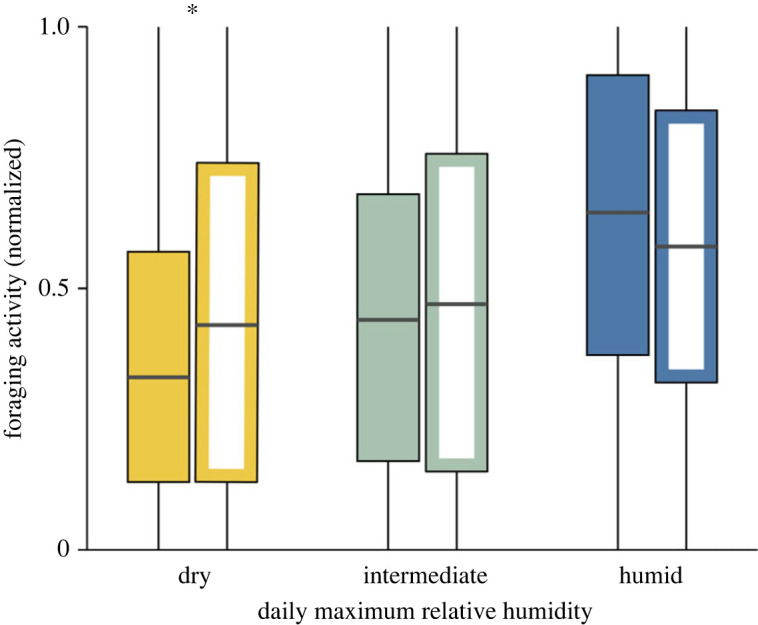
Comparison of foraging response to humidity level in colonies that reduce foraging on dry days and those that do not. Each bar represents the foraging activity, normalized for colony size as the proportion of the largest number of ants seen foraging in that colony in that year, on all days in all years with the indicated humidity level. Filled bars represent colonies that consistently reduce foraging on dry days; open bars represent colonies that do not. * = *p* < 0.05, Wilcoxon sign ranks test.

Over all days and all years of observation, the relation of foraging activity and daily maximum humidity differed in the two groups of colonies, those that reduced foraging on dry days in most years and those that never reduced foraging on dry days (figure 6). The slope of foraging activity versus humidity level differed in the two groups (*χ*^2^ = 7.8, *p* = 0.005). The same slope also differed among colonies within each group (*χ*^2^ = 6.6, *p* = 0.037). Foraging activity differed significantly in the two groups of colonies on dry days (Wilcoxon rank sum test, *W* = 53 164, *p* = 0.017). There were no significant differences between the two groups of colonies on days with intermediate humidity (*W* = 121 600, *p* = 0.345) or on humid days (*W* = 74 860, *p* = 0.095). There was a slight but not significant tendency for the colonies that reduced foraging on dry days to forage more on humid days (figure 6).

## 4. Discussion

Harvester ant colonies differ in the collective behavioural plasticity that manages water loss. Approximately 38% of colonies consistently reduce foraging activity, year after year, on days with low daily maximum humidity, while another 40% reduced foraging on dry days in some but not in most years. The rest, approximately 22% of the total, did not reduce foraging on dry days and at times even increased it.

This variation among colonies in the collective regulation of foraging activity arises from colony differences in individual forager response to olfactory interactions [39,41,51]. A forager's decision whether to leave the nest on its next trip depends on the rate at which it meets returning foragers, corresponding to food availability, as well as on humidity conditions [29]. Our results here suggest that colonies differ in how forager decisions depend on humidity, and that these responses are consistent from year to year.

It seems that differences among colonies in behavioural plasticity are responses to changes in humidity on the timescale of days. Colonies reduced foraging more on a dry day preceded by dry or intermediate conditions (e.g. 18 August 2018 or 21 August 2019), each preceded by two days with intermediate humidity, than when a dry day was preceded by humid days (e.g. 25 August 2021) (figures 1 and 2). Further data are needed to learn whether ants can retain enough water over several humid days to influence their foraging decisions if humidity then decreases.

How colonies adjusted foraging activity in dry conditions also varied from year to year (figures 2 and 5). Foraging activity is probably influenced by food supply [62], which changes from year to year as rainfall varies, influencing the production of seeds by annual plants. Since most of the seeds the ants eat are scattered by wind and flooding, the plants in a colony's location do not limit its food supply [32], and all colonies face similar limitations on food supply in a given year. Colonies store food at least for many months, and possibly for years; we have found seeds inside nests in summer that were produced the previous spring [32,63]. Colonies may differ in the amount of food stored, or in how the risk of water loss and colony hunger each influence forager activity.

While it is clear that foraging activity depends on humidity, we found no absolute threshold in humidity, common to all years, that determines when colonies tend to decrease foraging (figure 3). Further work is needed to learn exactly what are the environmental conditions, correlated with day-to-day changes in humidity, that the ants are responding to. Previous work suggested that colonies adjust activity in relation to the mean daily dew point, a measure that combines temperature and humidity [28], and here we found that colony response to mean daily dew point (figure 4) was similar to the response to other measures of humidity on the daily timescale.

Consistent differences among colonies in the regulation of foraging, year after year, reflect similar behavioural plasticity in successive cohorts of workers; different foragers of the same colony are alike in how their decisions to leave the nest depend on humidity. Previous work shows that within a colony, foragers respond similarly to current humidity [51]. There is no evidence that a colony's regulation of foraging in response to changes in humidity is due to its location on the 10 ha site, as the colonies that consistently reduce foraging on the site are distributed around the site, and colonies are responding to changes in relative humidity on a spatial scale much larger than the site. Colony survival depends on available foraging area, determined on the local density of neighbouring colonies, regardless of location on the site [36].

Plasticity in collective behaviour is ecologically important because it allows social animals to respond to changing conditions such as drought [64]. For harvester ants, variation among colonies in physiological and behavioural responses to thermal stress may be crucial in evolutionary rescue, as it is in other animals [65]. Early in the current drought in the southwest USA, which began in approximately 2000, harvester ant colonies that reduced foraging in dry conditions had higher colony reproductive success, in number of offspring colonies, than colonies that did not [25]. The drought has deepened since approximately 2003, and is now the most severe in 1200 years [66]. Colonies compete for foraging area in which to search for scattered seeds [36]. As the food supply has decreased, a colony requires more foraging area to survive [36]. If resources become more limited, colonies that reduce foraging activity in dry conditions may not be able to store enough food to survive, especially if they have neighbours that keep foraging high even on dry days.

Variation in behavioural plasticity provides the raw material for the evolution of behaviour [67–69]. The consistent behavioural plasticity in ant colonies reported here contributes to the growing evidence for collective behavioural plasticity in invertebrates (e.g. [70,71]), and more generally, to the evidence for shifts in the behaviour of social groups in response to environmental conditions (e.g. [72]). Collective behavioural plasticity, and its consequences for survival and reproduction, will help determine which populations can persist in a rapidly changing environment.

## Data Availability

Data are available from the Stanford Digital Repository at https://purl.stanford.edu/pp407bs8360 [73].
